# PARALLEL EVOLUTION OF LOCAL ADAPTATION AND REPRODUCTIVE ISOLATION IN THE FACE OF GENE FLOW

**DOI:** 10.1111/evo.12329

**Published:** 2013-12-23

**Authors:** Roger K Butlin, Maria Saura, Grégory Charrier, Benjamin Jackson, Carl André, Armando Caballero, Jerry A Coyne, Juan Galindo, John W Grahame, Johan Hollander, Petri Kemppainen, Mónica Martínez-Fernández, Marina Panova, Humberto Quesada, Kerstin Johannesson, Emilio Rolán-Alvarez

**Affiliations:** 1 Animal and Plant Sciences, The University of SheffieldSheffield S10 2TN, United Kingdom; 2 Biological and Environmental Sciences-Tjärnö, University of GothenburgSE-452 96, Strömstad, Sweden; 3 E-mail: r.k.butlin@sheffield.ac.uk; 4 Departamento de Bioquímica, Genética e Inmunología, Facultad de Biología, Universidade de Vigo36310 Vigo, Spain; 5 Departamento Mejora Genética INIACrta. La Coruña Km 7.5, 28040 Madrid, Spain; 6 Present address: LEMAR (UMR 6539), European Institute for Marine Studies, University of Western Brittany, Technopôle Brest-Iroise, rue Dumont d'Urville29280 Plouzané, France; 7 Department of Ecology and Evolution, University of ChicagoChicago, Illinosis, 60637; 8 School of Biology, University of LeedsLeeds LS2 9JT, United Kingdom; 9 Department of Biology, Lund UniversitySE-223 62 Lund, Sweden; 10 Faculty of Life Sciences, Manchester UniversityManchester M13 9PT, United Kingdom; 11 Present address: Molecular Oncology Unit, Basic Research Department, CIEMATAv. Complutense, 40, 28040 Madrid, Spain; 12 Department of Ecology and Evolutionary Biology, University of CaliforniaIrvine, 92697, California

**Keywords:** Gene flow, local adaptation, parallel evolution, speciation

## Abstract

Parallel evolution of similar phenotypes provides strong evidence for the operation of natural selection. Where these phenotypes contribute to reproductive isolation, they further support a role for divergent, habitat-associated selection in speciation. However, the observation of pairs of divergent ecotypes currently occupying contrasting habitats in distinct geographical regions is not sufficient to infer parallel origins. Here we show striking parallel phenotypic divergence between populations of the rocky-shore gastropod, *Littorina saxatilis*, occupying contrasting habitats exposed to either wave action or crab predation. This divergence is associated with barriers to gene exchange but, nevertheless, genetic variation is more strongly structured by geography than by ecotype. Using approximate Bayesian analysis of sequence data and amplified fragment length polymorphism markers, we show that the ecotypes are likely to have arisen in the face of continuous gene flow and that the demographic separation of ecotypes has occurred in parallel at both regional and local scales. Parameter estimates suggest a long delay between colonization of a locality and ecotype formation, perhaps because the postglacial spread of crab populations was slower than the spread of snails. Adaptive differentiation may not be fully genetically independent despite being demographically parallel. These results provide new insight into a major model of ecologically driven speciation.

Speciation is a central process in evolutionary biology. A growing consensus suggests that divergent natural selection in contrasting habitats, generating local adaptation, may be a common impetus for the evolution of reproductive isolation and thus speciation (Schluter 2009; Nosil 2012). The response to selection is straightforward in allopatry but both local adaptation and the subsequent enhancement of reproductive isolation may be opposed by gene flow and recombination where habitats are connected by dispersal (Felsenstein 1981; Smadja and Butlin 2011). Thus, although the traditional categorization of speciation processes into allopatric, parapatric, and sympatric classes may be an oversimplification (Butlin et al.2008), the spatial context for speciation and the extent of gene flow at different stages during speciation are still important in most scenarios, determining whether and how rapidly reproductive isolation will evolve (Butlin et al.2012). Understanding local adaptation and speciation therefore requires inferences about the biogeography and past demography of populations, factors that may have changed substantially over the course of speciation (Hewitt 2011; Abbott et al.2013). For example, speciation might be promoted by alternating cycles of separation by geographical barriers and secondary contact (Bierne et al.2011), or local adaptation might be achieved more readily with some spatial arrangements of habitats than with others (Gavrilets et al.2007). In principle, inferences about the sequence of events can be made using genetic data and coalescent-modeling approaches. For example, in the case of cave salamanders (*Gyrinophilus*), a model of continuous gene flow during divergence was supported over an alternative that allowed for a period of allopatry (Niemiller et al.2008; Pinho and Hey 2010). However, it was not possible to exclude short allopatric intervals and, in general, the reconstruction of complex gene-flow histories is expected to be challenging (Strasburg and Rieseberg 2013).

Cases of parallel local adaptation are of particular interest because they provide strong evidence for a role of natural selection. Where reproductive isolation repeatedly results from adaptation to similarly divergent pairs of environments, that is “parallel speciation,” this further shows that natural selection can drive speciation (Schluter and Nagel 1995). The natural replication provides the opportunity for powerful tests of underlying processes (Jones et al.2012). However, Johannesson et al. (2010) have emphasized that the pattern of parallel local adaptation in the presence of current gene flow can result from very different historical sequences of events. They distinguished four scenarios. Either the initial adaptive divergence occurred once, perhaps in allopatry, with subsequent colonization by differentially adapted forms of similar pairs of environments (scenario A) or, alternatively, evolutionary divergence occurred repeatedly in multiple localities, again with or without spatial separation (scenario B). Repeated evolution may depend on an independent origin of adaptive genetic variation in each population (B1), a common origin of locally adaptive alleles from standing genetic variation (B2) or concerted adaptation where each advantageous allele arose once and was then shared by gene flow between geographically separated populations in the same habitat (B3). Empirical separation of these alternatives requires, first, the use of putatively neutral genetic markers to establish the demographic history of the populations and, second, the analysis of loci underlying adaptation whose history may be substantially different from that for neutral markers (as for the *Eda* locus, Colosimo et al.2005, and other loci, Jones et al.2012, in sticklebacks). Key loci underlying local adaptation may be identified by genetic analysis (as for the *Eda* locus) or by “outlier” analysis (Stinchcombe and Hoekstra 2008). For outlier analysis, the first step of establishing the demographic history is essential because the reliable identification of loci under divergent selection requires a robust model of the demographic history of the populations analyzed (Crisci et al.2012).

Parallel origins for locally adapted ecotypes have often been invoked but have rarely been tested against explicit alternative hypotheses. Even in classic examples of colonization of lakes (Hohenlohe et al.2012; Kautt et al.2012) or caves (Strecker et al.2012) by fish, alternate histories are conceivable (Bierne et al.2013). Phylogenetic (Kautt et al.2012) or clustering (Strecker et al.2012) approaches do not contrast different models in the context of historical demographic change and gene flow. Here we test explicit alternative scenarios for the origin of parallel local adaptation in the rough periwinkle, *Littorina saxatilis*, a common rocky-shore gastropod from the North Atlantic that bears live young and has low lifetime dispersal (Reid 1996). In many locations, one finds two ecotypes in close proximity: a small, thin-shelled one with a large aperture, and a larger, thick-shelled form with a small aperture (Fig.1). These ecotypes are adapted to withstand wave exposure and crab predation, respectively (reviewed in Johannesson et al.2010). There is evidence for assortative mating, so that each morph mates preferentially with similar individuals (Conde-Padín et al. 2008), and for a genome-wide partial barrier to gene exchange (Grahame et al.2006), with evidence for divergent selection on some loci (Wilding et al.2001). The ecotypes (here referred to as “wave” and “crab” ecotypes) have been studied extensively, but to date largely independently, in three European regions: Galicia in northwest Spain (where the ecotypes are called “smooth unbanded” and “ridged banded,” respectively), the west coast of Sweden (“exposed” and “sheltered”), and the northeast coast of England (“high-shore” and “mid-shore”).

**Figure 1:**
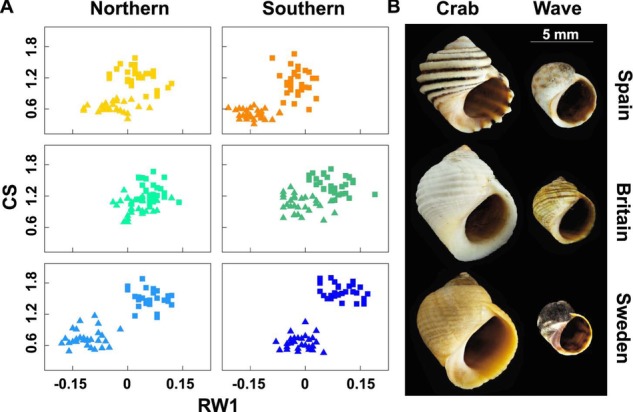
Parallel phenotypic differentiation. (A) Centroid size (CS) and the principal axis of shape variation (RW1) for specimens from each locality: wave ecotype—triangles, crab ecotype—squares. (B) Typical shells of each ecotype from the three regions studied (Photo: Fredrik Pleijel).

It has been suggested that ecotype differentiation occurred in parallel on different shores within Sweden and Spain (Johannesson et al.1993; Johannesson 2001; Rolán-Alvarez et al.2004; Panova et al.2006; Quesada et al.2007), and this has been widely accepted (Ostevik et al.2012), although the evidence has been questioned (Butlin et al.2008). Furthermore, parallel differentiation at the level of regions (i.e., Britain, Sweden, Spain) has not previously been tested: it is possible that the ecotypes originated independently in different parts of Europe but that there was a single origin within each region. Recent phylogeographic analyses (Doellman et al.2011; Panova et al.2011) suggest that Iberian populations have been genetically independent from northern European populations for a longer period than the separation of Swedish from British populations, which are likely to have shared a postglacial colonization history. Here we analyze samples from all three regions together, for the first time. We test for parallel adaptation by asking to what extent ecotypes have diverged in the same phenotypic direction between regions and between sites within regions. We then combine mitochondrial and nuclear DNA sequence and amplified fragment length polymorphism (AFLP) data, using an Approximate Bayesian Computation (ABC) framework (Beaumont 2010; Wegmann et al.2010), to compare models for the demographic history of the populations. Our question is this: was the origin of the ecotypes a single event or a series of parallel events, occurring either within each locality or within each European region? Our results provide strong support for parallel demographic separation at both spatial scales.

## Methods

### SAMPLING

Snails were sampled between January and November 2008 from six localities. Two localities were sampled per region separated from each other by 65 km in Sweden, 266 km in Britain, and 290 km in Spain: Tjärnö (Long. +58°49′26″, Lat. +11°3′46″) and Lysekil (+58°16′12″, +11°24′55″) in Sweden, Dunbar (+56°00′20″, −2°30′42″) and Thornwick (+54°07′57″, −0°06′54″) in Britain and Burela (+43°40′37″, −7°22′5″) and Silleiro (+42°6′45″, −8°53′58″) in Spain. At each locality, we collected in both the “wave” and the “crab” habitats, spacing samples along the shore to avoid collecting related individuals. We worked with females to avoid confusion with the related species *Littorina arcana*, in which males are indistinguishable from those of *L. saxatilis* (Reid 1996). Thirty-two females per ecotype were used, separated by 1 m intervals wherever possible.

### MORPHOMETRIC ANALYSIS

Each snail was photographed with a Leica MZ12 stereoscopic microscope and Leica digital ICA video camera. The presence of shell scars was noted, indicating that the individual had survived a crab attack (Vermeij et al.1981; Johannesson 1986). We predicted that scars would be more frequent in the “crab” habitat because of a greater probability of both attack and survival. Adult shell images (*n* = 26–30 per ecotype per location) were analyzed using 11 landmarks positioned on the digitized shell image following Conde-Padín et al. (2009).

For each individual, we measured centroid size (CS) and shape, using relative warps (RW). The relative warps were computed using the software packages TpsDig and TpsRelw (Rohlf 2005, 2006), excluding the uniform component, following Carvajal-Rodríguez et al. (2005). We used the scaling option α = 0, which weights all landmarks equally.

We performed a three-way analysis of variance (ANOVA) on size and shape variables, with fixed factors region (Spain, Britain, and Sweden) and ecotype (Wave, Crab), and locality as a random factor nested within the interaction between fixed factors. We used a G-test to compare scar frequencies between ecotypes and regions.

### DNA EXTRACTION AND SEQUENCING

Head–foot tissue was used for DNA extraction, using a CTAB protocol (Wilding et al.2001). DNA concentration and purity were assessed using a NanoDrop spectrophotometer. DNA samples were purified with NucleoSpin columns following the manufacturer's instructions (Macherey-Nagel). All DNA samples were standardized to 50 ng·μL^−1^.

Primers designed from the annotated *L. saxatilis* partial mtDNA sequence (AJ132137; Wilding et al.1999) and from sequences in Small and Gosling (2000) were used to amplify a 2004bp region (in two overlapping fragments of 1028 and 1137bp) encompassing the *ND6* and *tRNApro* mitochondrial genes, as well as the 3′ end of the *ND1* gene and the 5′ end of the *Cyt-b* gene (Table S1). Sixteen individuals were sequenced for each ecotype in each locality.

The candidate nuclear genes were chosen from the *Littorina* Sequence Database (Canbäck et al. 2012). Three exon-primed intron-crossing (EPIC) markers were successfully designed, targeting a complete intron of the calreticulin (*Cal*), elongation factor 1 α (*ElFac*), and thioredoxin peroxidase 2 (*ThioPer*) genes (Table S1). Each gene was amplified in 16 individuals per ecotype from each locality, polymerase chain reaction (PCR) products were cloned and inserts sequenced (see Table S1 for details).

### SEQUENCE ANALYSIS

For mtDNA, contigs were assembled using Seqman (DNASTAR, Madison, WI), aligned with ClustalX (Thompson 1997) and inspected in BioEdit 7.0.9.0. (Hall 1999). Haplotypes were obtained with Collapse 1.2 (available from http://darwin.uvigo.es). Polymorphism estimates, neutrality tests, and other population genetic analyses were conducted in DnaSP 4.0 (Rozas et al.2003) or Arlequin version 3.5.2 (Excoffier and Lischer 2010). To obtain summary statistics for the ABC analysis, we used the Kimura 2-parameter model for consistency with our simulated data. A haplotype network was built with Network 4.6.1.0 (Bandelt et al.1999).

Sequence data for nuclear loci were processed using the software Geneious Pro version 5.1.7 (Biomatters Ltd., Auckland, New Zealand). Primers and vector were trimmed and sequences were aligned with the clustalw2 algorithm (Larkin et al.2007). All sequences were inspected at every polymorphic position to detect possible sequencing errors.

The distinct alleles present in each alignment were identified with Arlequin. For each gene, the occurrence of no more than two alleles per individual was checked to identify PCR artifacts. Doubtful individuals were first amplified, cloned and sequenced a second time, and ultimately discarded if ambiguities could not be resolved. Finally, only one allele was kept per individual and per gene. For all further analyses, the exonic regions were trimmed and all indels were deleted. The final alignments were composed of 192, 187, and 189 sequences of length 376, 314, and 341bp, respectively for *Cal, ElFac*, and *ThioPer*.

The haplotype and nucleotide diversities were estimated in Arlequin. To obtain summary statistics for the ABC analysis, we used the Kimura 2-parameter model for consistency with our simulated data. The neutrality of the nuclear loci was verified with the Tajima's *D* and Fu's *F*s tests, implemented in Arlequin, and the significance was assessed with 10,000 simulations. A sequential Bonferroni correction for multiple tests was applied to the neutrality tests. In addition, a recombination test was performed for each gene with IMgc Online (Woerner et al.2007). Recombination was detected only for *ElFac* and the first 44bp at the 5’ extremity of the intron were removed from the alignment to exclude possible recombining sites in further analyses. Haplotype networks were built with TCS version 1.21 (Clement et al.2000) and edited with Inkscape 0.48.1 (www.inkscape.org).

Nuclear sequence data have been submitted to GenBank with accession numbers: *Cal* HG792757–HG792783, *ElFac* HG792716–HG792756, and *ThioPer* HG792699–HG792715. The mtDNA fragment corresponds to GenBank Accession AJ132137, starting at position 710. A haplotype file for individuals studied here is available at Dryad: doi:10.5061/dryad.m186r.

### AMPLIFIED FRAGMENT LENGTH POLYMORPHISMS

All 32 individuals per locality and ecotype were used in AFLP analysis. Profiles were generated with two rare-cutter enzymes (*Eco*RI and *Pst*I) to minimize homoplasy (Caballero et al.2008). The method was based on Vos et al. (1995). A double digestion with 2U of *Eco*RI and *Pst*I (New England Biolabs, Ipswich, MA) was carried out in a final volume of 11.6 μL 1× Eco buffer (New England Biolabs) containing 100 ng of genomic DNA and 3 μg of bovine serum albumin. Samples were digested for 3 h at 37°C. Then, 5.5 μL of 1× ligation buffer (Invitrogen, Carlsbad, CA) containing 0.5U of T4 DNA ligase (Invitrogen), 0.9 μM Eco-adaptor, and 0.9 μM Pst-adaptor were added to the digestion reaction. Samples were ligated for 16 h at 16°C. Ligation reactions were diluted 1:4 and used as template for preselective PCRs. Six different selective PCRs were performed and 12 primer combinations were obtained (Table S1). Preselective and selective PCR conditions, electrophoresis, and scoring are described in Galindo et al. (2009) (and see Supporting Information). AFLPscore version 1.4 (Whitlock et al.2008) was used to perform error rate analysis using replicates (15% of the samples, chosen randomly and replicated from the digestion step), remove loci with low repeatability, and create the binary matrix (0, 1) containing the AFLP phenotypes. The mismatch error rate obtained with AFLPscore was 4.63% and the number of AFLP loci scored was 614. The AFLP dataset has been submitted to Dryad: doi:10.5061/dryad.m186r.

Outlier analysis was performed using the program Dfdist (Beaumont and Nichols 1996; http://www.maths.bris.ac.uk/∼mamab/stuff/). Dfdist input files were created using the AFLP convert program (http://webs.uvigo.es/acraaj/tools.htm) and analyses were carried out following Galindo et al. (2009). Between-ecotype pair-wise comparisons were performed independently for each locality and those loci above the 95th percentile were considered outliers. Outliers within locality were combined for each region to determine the degree of sharing of outliers, because sharing is one possible indication of the repeated involvement of the same loci in the response to divergent selection. All the outliers detected in any of the six localities were removed from the dataset in further analyses because demographic parameters are best estimated using exclusively loci that are not influenced by selection (Beaumont and Nichols 1996). After removing outliers AFLP-SURV version 1.0 (Vekemans et al.2002) was used to calculate *F*_ST_ values and individual pair-wise relatedness, which was used to create multidimensional scaling plots, following the methodology of Jackson et al. (2012). Summary statistics for ABC analysis were calculated using the same custom scripts as we used for simulated data. The analysis of molecular variance (AMOVA) was conducted in Arlequin.

### APPROXIMATE BAYESIAN COMPUTATION

#### Demographic models and parameters

Models are specified in Figure3. There were 8(9) parameters of interest for the parallel (old divergence) models, either within or between regions, plus two mutation parameters: MU, mutation rate for nuclear sequence data; AFLPMU, mutation rate for AFLP sequences. Observed summary statistics are given in Table S2.

We conducted exploratory simulations to ensure that the prior distributions for our demographic parameters encompassed the posterior distributions, while remaining biologically reasonable. Where parameters were shared between models, we used the same prior distribution. In the case of equivalent parameters between models (e.g., in the parallel divergence model the first time split, between ecotypes, is equivalent to the first time split between localities in the ancestral divergence model), we also used equal prior distributions. Prior distributions were log uniform except for mutation rates and time proportions (PROPT, etc.), which were uniform, and ranges are given in Table S3.

We set the mutation rate for mitochondrial loci to 1.5 × 10^−8^ per base per generation based on a substitution rate of 3% per million years from the fossil record for *Littorina* species (after Reid et al.1996; Wares and Cunningham 2001) as used by others (Wares et al.2002; Blakeslee et al.2008; Chapman et al.2008; Cunningham 2008). For the three sequenced nDNA loci, we allowed the mutation rate to vary over one order of magnitude below the mitochondrial mutation rate: 1.5 × 10^−8^ to 1.5 × 10^−9^ per base per generation. For AFLP loci, we allowed the mutation rate to vary independently but over the same range as the nuclear loci. For the mtDNA sequence, we used a transition/transversion ratio of 0.91, based on third position cytochrome *b* data from Reid et al. (1996). For the nDNA sequences, we used an unbiased transition/transversion ratio of 0.33.

Because we conducted 10^6^ simulations per model, and used the same simulation set to test multiple individual observed datasets in some cases, we set the simulated sample sizes for the coalescent simulations as the geometric mean of the real sample sizes across the ecotypes/localities used as observed datasets.

#### ABC sampling

For each model, we performed 10^6^ standard ABC simulations using the software package ABCtoolbox (Wegmann et al.2010) for all markers combined. For the within-regions models, we also performed 10^6^ simulations separately for the sequence data and for the AFLP data.

We used *fastsimcoal* (Excoffier and Foll 2011) to simulate sequence data for four loci with lengths equal to the observed sequences (after pruning of *ElFac* to remove putative recombinants), and *arlsumstat* to calculate summary statistics. For the AFLP loci, we used *fastsimcoal* to simulate 462 separate loci, each one with a 20 base sequence, and an in-house program that converted the resulting sequence data into a binary matrix of AFLP alleles and calculated summary statistics from this binary matrix. For each simulated AFLP locus, one 20bp haplotype was chosen at random and designated the “1” allele. All other haplotypes were designated as “0” alleles. AFLP phenotypes were then called assuming that genotypes 11 and 10 correspond to “band present” and genotype 00 corresponds to “band absent.” This process allowed us to simulate the asymmetrical mutation expected for presence and absence alleles at AFLP loci and the fact that loci fixed for the absence allele are not observed. Calculating summary statistics from the simulated phenotype matrix made them directly comparable to the summary statistics obtained from the real data.

#### Summary statistics and estimation step

Summary statistics used for the sequence data (for each sample or sample pair) were Tajima's *D*, π, and Φ_ST_ based on Kimura 2-parameter distances between sequences, implemented in Arlequin/arlsumstat 3.5. For the AFLPs, we used heterozygosity, mean *F*_ST_, standard deviation of *F*_ST_ across loci, and Jaccard distance. Because our summary statistics were numerous (56 for the sequence data; 22 for the AFLP data; 78 when combined), we used the partial least squares (PLS) method described in Wegmann et al. (2009) to reduce their dimensionality in the rejection step of the ABC procedure (see Supporting Information).

Separate sets of PLS components were defined for simulations under each different model because of the variation in parameters. These PLS components were used to transform the summary statistics of the entire dataset of simulations, as well as the observed summary statistics, prior to the estimation stage. After retaining the closest 0.5–2% of simulations to the observed data based on PLS components, we took two approaches for the “regression adjustment” step, depending on whether we were interested in model comparison or parameter estimation. For model comparison, we used all summary statistics to perform postsampling adjustment, using the GLM method of Leuenberger and Wegmann (2010), to produce marginal densities, which were comparable between models. For parameter estimation, we used PLS components for both the distance step and the postsampling adjustment step.

For combined datasets, we retained the closest 10,000 simulations (1%) to the observed data based on the Euclidean distance between PLS-transformed observed summary statistics and PLS-transformed simulated summary statistics, and used these retained summary statistics to estimate the parameter values that best reproduce the real-world data using the ABC-GLM procedure of Leuenberger and Wegmann (2010), implemented in ABCtoolbox. For the AFLP data, we took the same approach, but retained the closest 20,000 simulations to the observed data. For sequence data alone, we retained the closest 5000 simulations to the observed dataset. These numbers of retained simulations were chosen on the basis of the *P*-values (the fraction of retained simulations with likelihood less than or equal to the likelihood of the observed data under the GLM).

#### Model comparison and validation

Models were compared using Bayes Factors, which are equal to the ratio of the marginal densities between models, and posterior probabilities, which are approximately equal to the marginal density of the model of interest divided by the sum of the marginal densities of all models. The *P*-value was used as a measure of goodness-of-fit.

In addition to these tests, we also compared the distribution of summary statistics of retained simulated datasets to the summary statistics of the observed dataset, to check that the observed dataset lay well within the distribution of simulations. We did this for distributions of both PLS (all pairs of variables) and raw summary statistics (one variable at a time). The condition was satisfied for all reported models.

To validate our model choice, we simulated 1000 (new) datasets from the original priors for each competing model and used these pseudo-observed datasets to test the robustness of discrimination between models (Fig. S2). To validate our parameter estimates, we used the 1000 pseudo-observed datasets that were generated under each model to check for uniformity of the posterior quantiles (Fig. S3).

## Results

Morphometric analysis of *L. saxatilis* shell size and shape, from two sites in each of three regions, showed remarkable concordance in the direction of phenotypic differentiation between samples from crab- and wave-dominated habitats (Fig.1). Despite some differences between regions, crab ecotype snails were consistently larger and had higher scores on the first shape axis (RW1; Table1), representing a smaller aperture and higher spire than wave ecotype snails. Differentiation between ecotypes was most marked in Sweden and least marked in Britain. Previous studies, in both Sweden and Spain, indicate that the majority of the morphological difference between ecotypes is genetically determined, although there is a small contribution from developmental plasticity (Janson 1982; Johannesson and Johannesson 1996; Conde-Padín et al. 2009; Saura et al.2012). We predicted that snails in the crab-exposed habitat would be attacked more often by crabs and also be more likely to survive attacks, resulting in a higher frequency of scarred shells. As expected, the crab ecotype had a higher proportion of snails with scars than the wave ecotype (42.2% vs. 20.0%, *G* = 21.6, df = 1, *P* < 0.001). This difference was also greatest in Sweden (Table2).

**Table 1:** Three-way ANOVA for the morphometric variables of centroid size (CS) and shape (the two leading relative warp axes, RW1 and RW2). The percentage of variance explained by each relative warp is presented in parenthesis). We checked for heteroscedasticity in the dependent variables; CS did depart from expectation and so results for this variable should be taken with some extra caution

Trait	Source	DF	MS	*F*	*P*	*R*^2^
CS	Ecotype	1	31.79	16.04	0.0571	47.3
	Region	2	3.48	1.76	0.3626	26.9
	Interaction	2	1.98	11.34	0.0091	18.9
	Locality (interaction)	6	0.17	6.72	9.9 × 10^−7^	6.9
	Error	333	0.026			
RW1	Ecotype	1	0.856	15,08	0.0046	35.9
	Region	2	0.414	4.62	0.1779	26.2
	Interaction	2	0.089	0.74	0.5731	9.04
(56.6%)	Locality (interaction)	6	0.365	44.26	1.1 × 10^−39^	28.1
	Error	333	0.457			
RW2	Ecotype	1	0.037	2.83	0.2340	26.7
	Region	2	0.015	0.58	0.6320	11.5
	Interaction	2	0.026	1.32	0.3343	19.3
(16.6 %)	Locality (interaction)	6	0.059	7.63	1.1 × 10^−7^	42.5
	Error	333	0.432			

**Table 2:** Frequency analysis for presence/absence of shell scars. Scars indicate specimens that survived a crab attack

			Number	Number
			with	without
Region	Locality	Morph	scars	scars
Sweden	North (Tjärnö)	Crab	15	11
		Wave	6	23
	South (Lysekil)	Crab	18	11
		Wave	7	23
Britain	North (Dunbar)	Crab	13	14
		Wave	9	19
	South (Thornwick)	Crab	11	18
		Wave	1	29
Spain	North (Burela)	Crab	10	20
		Wave	11	17
	South (Silleiro)	Crab	7	23
		Wave	1	28
All regions		Crab	74	97
		Wave	35	139

Mitochondrial DNA sequence data showed extensive sharing of haplotypes between British and Swedish localities but not between these regions and Spain (Fig. S1; AMOVA by region: Φ_CT_ = 0.14, *P* = 0.037). The northern locality in Spain (Burela) showed much higher diversity than the southern locality (Silleiro) whereas all other localities showed diversity similar to Burela. In no case was there strong differentiation between ecotypes (Table S2) and ecotypes were not differentiated overall (AMOVA by ecotype: Φ_CT_ = 0). These patterns are consistent with previous observations (Quesada et al.2007; Doellman et al.2011; Panova et al.2011) and suggest genetic isolation between northern European and Spanish populations as well as either a recent origin of ecotypes, or substantial gene flow between them within localities.

Sequence data from introns in three single-copy nuclear genes (*ElFac, Cal, ThioPer*; 314, 376, and 341bp, respectively) revealed lower overall diversity than mtDNA (Fig. S1). Neutrality tests did not reveal departures from expectations for any of the three introns in any locality. As for mtDNA, there was evidence for differentiation between regions but not between ecotypes (AMOVA by region: Φ_CT_ = 0.64, 0.16, and 0.19 for *ElFac, Cal*, and *ThioPer*, respectively, *P* ≤ 0.009; by ecotype: Φ_CT_ = 0 for all loci).

After quality checking and removal of loci with poor repeatability, the AFLP dataset included 614 loci. We excluded 152 of these loci that showed evidence for an influence of divergent selection between ecotypes within any locality. Fewer of these outliers were observed in Britain, where morphological differentiation was also less marked than in the other regions. There was slightly greater sharing of outliers between localities and between regions than expected by chance (Table4). As in previous analyses of British (Wilding et al.2001) and Spanish (Galindo et al.2009) populations, differentiation between ecotypes within localities was low (*F*_ST_ = 0–0.027) relative to differentiation among localities (*F*_ST_ = 0.021–0.134), and the highest genetic distances were between Spanish and northern European localities (*F*_ST_ = 0.107–0.134). As for the other marker types, overall differentiation was strong among regions but not between ecotypes (AMOVA by region: *F*_CT_ = 0.132, *P* < 0.001; by ecotype: *F*_CT_ = 0).

**Table 3:** *G*-test decomposition for frequency of shells with scars

Region	Locality	*G*_morph_	DF
All pooled		21.6***	1
	Between regions	5.4	2
Sweden	Pooled	17.4***	1
	Between	0	1
	North	7.9**	1
	South	9.1**	1
Britain	Pooled	9.0**	1
	Between	4.3*	1
	North	1.4	1
	South	11.8***	1
Spain	Pooled	0.8	1
	Between	4.6*	1
	North	0.2	1
	South	5.2*	1

* *P* < 0.05.

** *P* < 0.01.

*** *P* < 0.001.

**Table 4:** Summary of the AFLP outlier analyses with Dfdist based on 614 loci. Six pair-wise comparisons were carried out between ecotypes, within localities. Average *F*_ST_, 30% trimmed *F*_ST_, and average *F*_ST_ of the simulations are shown. Sample sizes (*N*) of each ecotype within locality are also shown. “N outliers” represents the number of outliers (95th percentile) detected in each locality and “Overall” is the total number of distinct outlier loci combining the results from all the localities. “Shared outliers” are loci that were detected in two localities or regions. Expected values for shared outliers are simply based on the observed proportions in each separate analysis

		Sample size	Average	Trimmed	Simulated	*N*	Shared outliers (observed/expected)		
Region	Locality	(crab/wave)	*F*_ST_	*F*_ST_	*F*_ST_	outliers (95%)	Within region	Sweden	Britain
Sweden	Tjarno	26/25	0.0517	0.0070	0.0125	51			
	Lysekil	28/25	0.0679	0.0291	0.0337	28	8/2.3		
Britain	Dunbar	24/17	0.0067	−0.0125	0.0092	11			
	Thornwick	20/20	0.0302	0.0006	0.0088	21	1/0.4	6/3.6	
Spain	Burela	29/24	0.0952	0.0318	0.0363	42			
	Silleiro	27/27	0.0639	0.0216	0.0270	36	4/2.5	15/8.6	4/3.7
Overall						152			

**Figure 2:**
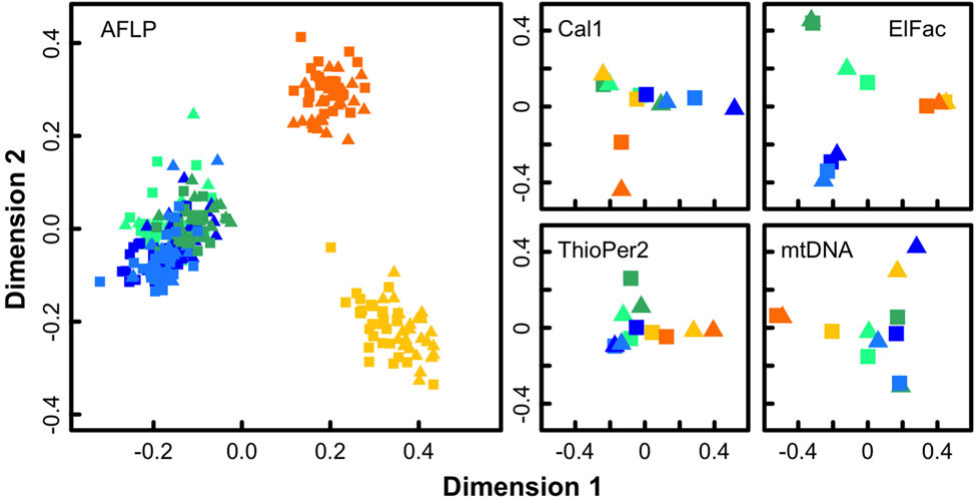
Multidimensional scaling plots for the AFLP data (left, using individual relatedness) and each of the sequence datasets (right, using Φ_ST_ between samples). Symbols: wave ecotype—triangles, crab ecotype—squares; Spain—orange, Britain—green, Sweden—blue symbols; Northern site—light shade, Southern site—dark shade.

**Figure 3:**
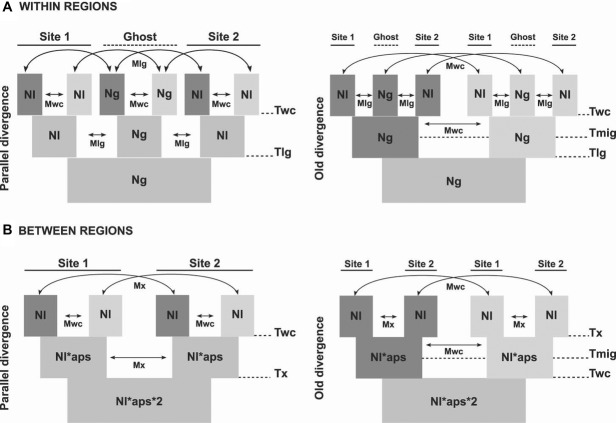
Historical demographic models compared using ABC. (A) Two sites within the same region. (B) Two sites, each in a different region. Dark shading indicates Crab ecotype, light shading the Wave ecotype and intermediate shading the ancestral populations. 1, 2—sampled localities; arrows signify migration. The present is represented at the top of each diagram.Parameters used were: Within-region models: Ng – effective size of the ancestral and ghost population, Nl – effective size of a local population, Tlg – time of separation of the spatially separated populations, Twc – time of separation of the ecotype populations, Tmig (old divergence model only) – time since the end of allopatric separation of ecotypes, PROPT – (parallel model only) – log(Twc)/log(Tlg), PROPTlg (old divergence model only) – log(Tlg)/log(Twc), PROPmig (old divergence model only) – log(Tmig)/log(Twc), Mlg – probability of an individual migrating between a local and a ghost population (no direct migration allowed between local populations), Mwc – probability of an individual migrating between populations of the different ecotypes. For the old divergence model, the constraint PROPmig>PROPTlg was imposed. Between-region models: Nl – effective size of a local population, APS – relative size of the ancestral population, Tx – time of separation of the regional populations, Twc – time of separation of the ecotype populations, Tmig (old divergence model only) – time since the end of allopatric separation of ecotypes, PROPT (parallel model only) – log(Twc)/log(Tx), PROPTx (old divergence model only) – log(Tx)/log(Twc), PROPmig (old divergence model only) – log(Tmig)/log(Twc), Mx – probability of an individual migrating between populations in different regions, Mwc – probability of an individual migrating between populations of the different ecotypes. For the old divergence model, the constraint PROPmig>PROPTx was imposed.

There were some common patterns among the marker types, particularly the low differentiation between ecotypes compared with the separation among regions, but also many differences of detail (Fig.2) as expected from the stochasticity of the underlying processes of mutation, drift, and gene flow. A key question is whether the low differentiation between ecotypes within localities is because of recent parallel origin of the ecotypes *in situ* in each region or locality, or to a single, older common origin whose genetic signal has been obscured by subsequent gene exchange. Ideally, data from all markers should be combined to answer this question. Therefore, we formalized the two alternative models (Fig.3) and compared them using ABC. In the “parallel divergence” model, an ancestral population colonized multiple localities, which were connected by gene flow, and the ecotypes then diverged within localities creating a partial barrier to gene flow whose effects may be detectable in neutral loci. We considered periods of allopatry (zero gene flow) between ecotypes, within localities to be biologically implausible and so did not include them in this model. In the “old divergence” model, ecotypes diverged within the ancestral population before colonization of the sampled localities, potentially with an initial period of allopatry.

First, we applied these models to the two sampled localities within each region (Fig.3A). In each case, “ghost” populations (Beerli 2004) were included in the model to represent the many unsampled localities that make up the regional meta-population. The parallel divergence model was strongly supported relative to the old divergence model for British and Spanish populations (posterior probabilities 0.9974 and 0.9965, respectively) but the opposite was true for the Swedish populations (posterior probability of 1.0000 in favor of the old divergence model). Nevertheless, a model where the period of allopatry was constrained to be zero had a higher posterior probability for the Swedish populations. Thus, there was evidence for continuous gene flow even where a single origin of the ecotypes was supported. We also considered sequence (nuclear+mtDNA) and AFLP data separately. Sequence data gave the same pattern as the combined data but AFLPs supported the parallel divergence model for all regions, including Sweden. The preferred models (parallel for Britain and Spain, old divergence for Sweden) fitted the data well: observed summary statistics fitted the ABC regression estimation (*P* > 0.17; Table S3) and fell within the range of postrejection simulated values (both untransformed and PLS), and the distributions of posterior quantiles did not show strong departures from uniformity, indicating a lack of bias in parameter estimation (Wegmann et al. 2009). The alternative models both allow the possibility of gene flow connecting all populations for most of their history and, therefore, they are likely to be difficult to distinguish. Nevertheless, pseudosamples generated under the alternative models, using prior distributions of parameters, showed that discrimination between models was possible using the combined dataset since for 54% of pseudosamples the correct model was supported with confidence (for 21% the wrong model was supported and in the remainder neither model had posterior probability >0.95; Fig. S2; see Materials and Methods for details of model validation).

Parameter estimates for the within-region models using data for all markers had wide 95% highest posterior density intervals (Table S3). However, the median estimates from the parallel divergence model were consistent across regions in suggesting large effective population sizes (∼10^4^ locally and ∼10^6^ for the ghost populations), low migration rates between localities (*m*∼10^−5^), and long times since the first population separation ∼10^5^ generations (there are typically 2 generations per year). The parallel divergence models estimated the time since ecotype formation to be ∼10^4^ generations, that is with long times between colonization and ecotype formation, and low migration between ecotypes (∼10^−6^ per generation). These estimates are referenced to a fixed mtDNA mutation rate (see Materials and Methods).

To investigate ecotype origin at the regional level, we applied the same two models to the combined marker data for each of the possible between-region pairs of localities in turn (Fig.3B). We did not include ghost populations because, in this case, we took the locality sample to be representative of its regional meta-population. In 11 of 12 comparisons, the parallel divergence model was strongly supported (posterior probability >0.9996; Table S3). As for within-region models, observed summary statistics fitted the ABC regression well (*P* = 0.097–0.282, the lowest value being for the Lysekil–Thornwick pair for which the old divergence model was preferred). The robustness of discrimination between models was slightly greater in this case (probability of supporting the correct model 59% and the wrong model 17%; Fig. S2). In the parallel divergence model, local population sizes were again estimated to be ∼10^4^, except where the southern Spanish Silleiro site was involved where estimates were greater (∼10^5^). These models allowed the possibility of population expansion associated with ecotype formation but the posterior distributions did not support this (Table S3). Migration estimates between ecotypes were similar to the within-region estimates (∼10^−6^ per generation) but migration between sites in different regions was, as expected, estimated to be lower (10^−6.5^–10^−8^), with the lowest values involving the Silleiro site which appears be more distinct from the northern regions than the other Spanish site (Table S3). Averaging parameter estimates across comparisons (excluding Lysekil–Thornwick), the between-region models suggest separation of British and Swedish populations ∼6 × 10^5^ generations ago and separation of northern from Spanish populations ∼1.4 × 10^6^ generations ago, whereas ecotype separation is estimated to be much more recent (∼5 × 10^3^ generations from Britain-Sweden comparisons and ∼2.6 × 10^4^ generations from northern Europe–Spain comparisons), as for the within-region models (∼10^4^ generations).

## Discussion

Our results show strikingly parallel phenotypic differentiation in *L. saxatilis* in response to contrasting crab and wave environments, both between localities within regions and among European regions. Our analyses of genetic data support the hypothesis that the ecotypes arose in parallel, without allopatric separation and after colonization of the different regions and localities, rather than divergence being old and predating colonization. This support depends on the effect on neutral loci of the barriers to gene flow between populations in the contrasting environments that are associated with phenotypic differentiation. It remains to be seen whether the alleles underlying adaptive traits evolved in parallel.

Our data confirmed previous observations, based on mtDNA, of greater differentiation between Spain and northern Europe than between Britain and Sweden and of a large genetic distance between the two Spanish sites (Quesada et al.2007; Doellman et al.2011; Panova et al.2011). Reid et al. (1996) used fossil and biogeographic information to provide estimates of evolutionary rates. Our parameter estimates depend on their mtDNA mutation rate but we adjusted the mutation rate to fit the simple substitution model implemented in the ABC analyses. For this reason and because the long-term rate may not be appropriate for the recent events described here (Charlesworth 2010), the inferred relative timings of events and population sizes may be more robust than absolute values. Relative values are consistent with the interpretation of postglacial colonization of British and Swedish shores from a common refuge or refugia (Doellman et al.2011; Panova et al.2011), distinct from the Spanish sites, because the estimated time of separation between the northern European regions and Spain was 2.2 or 7.4 times older than between Britain and Sweden (based on the between-region and all-region models, respectively). Two separate refugia in Spain can be inferred from the estimated time of separation between sites, which was about 10 times greater than in the northern regions.

Our models gave little support to past population expansion, even suggesting a reduction in population size in Spain. Doellman et al. (2011) and Panova et al. (2011) found some evidence that *L. saxatilis* population sizes had expanded recently using their mtDNA markers (∼10×). They inferred large population sizes and, as in our analyses, their IMa model fits implied gene flow between geographical regions. As in Panova et al. (2011), our between-region models implied about 10 times more gene flow between Britain and Sweden than between these regions and Spain. Overall, the evidence suggests that the distribution of *L. saxatilis* changed during the Pleistocene glacial cycles but that the effective population size remained consistently large, partly as a result of gene flow over large distances. *Littorina saxatilis* is cold-tolerant and now has a distribution extending into the Arctic. Therefore, its populations may not have been impacted as severely as some species by the glaciations, resulting in early colonization of northern European shores with limited population expansion and allowing long-term separation from southern European populations.

Our analyses support the parallel divergence of ecotypes over the alternative old divergence model (Fig.3), that is they suggest that the crab and wave ecotypes arose separately in each region and locality, after colonization, rather than arising once before the geographical separation of populations. This is true both within and between regions. The confidence that can be placed on model comparisons in ABC has been questioned (Robert et al.2011) because of the uncertainty introduced by the choice of summary statistics. Therefore, we used an information-rich set of summary statistics, derived from different marker types, and used all summary statistics for model comparison (Leuenberger and Wegmann 2010). All preferred models fitted the observed data well and in all cases the distributions of simulated summary statistics, after the rejection step, contained the observed summary statistics. Extensive recent gene flow may eradicate signals of past separation (Bierne et al.2013) making our alternative models intrinsically difficult to distinguish, at least for part of the parameter space. However, our model choice validation indicated that discrimination between the parallel and old divergence models was possible, with reasonable support. At least for between-region comparisons, the consistent evidence in favor of the parallel model (11 of 12 comparisons) is very unlikely to be a chance outcome.

Under the parallel model, the separation of ecotypes was in every case estimated to be recent relative to the separation of populations in different localities (itself presumably reflecting patterns of colonization). In the within-regions models, the time to ecotype separation was about 10% of the age of the local populations whereas for between-region models it was an even smaller fraction (1–2%), as expected. If the actual time of colonization of British and Swedish coasts was after the most recent glacial retreat (∼10,000 years ago; Charbit et al.2007), the relative age of ecotype separation implies a waiting time to ecotype formation of around 18,000 generations (9000 years). Absolute time estimates from the models imply even longer waiting times. This contrasts with simulation results for ecotype formation in *Littorina* (Sadedin et al.2009), based on the characteristics of the Swedish populations, in which distinct morphs form rapidly (typically in <1000 generations). Models of ecological speciation generally have shorter waiting times to speciation than models that do not involve direct divergent selection but waiting times are dependent on the supply of relevant mutations (Gavrilets 2004). In our demographic models, ecotype formation occurs instantaneously rather than gradually. This may be considered unrealistic but intermediate levels of differentiation occur only briefly in the Sadedin et al. (2009) models. Barriers to gene flow at neutral loci are never strong in these models (*F*_ST_ reaches about 0.05) but those barriers that do also appear rapidly.

Two possible explanations for ecotype formation occurring long after colonization of each locality deserve further investigation. The major predators considered important in selecting for the “crab” ecotype may have arrived in warming regions after the snails because they require higher minimum temperatures. *Carcinus maenas* (predator in Britain and Sweden) has a current northern distribution limit well south of the northern limit of *L. saxatilis*, whereas *Pachygrapsus marmoratus* (predator in Spain) is a relatively warm-water species. Alternatively, following local extinctions (due to toxic algal blooms, e.g.; Johannesson and Johannesson 1995) populations may be reestablished by individuals that bring (neutral) alleles from source populations of both morphs. Our model fits for the time of separation of ecotypes may then reflect these recent events rather than the original ecotype formation.

The parallel model involves an ancestral population that became divided spatially into a series of local populations, which exchanged migrants. Later, distinct habitat-associated populations were established within each of these local populations, still with gene exchange. This scenario was clearly favored over the old divergence model for both British and Spanish populations but the support was equivocal for Swedish populations. Biologically, what does support for the parallel model mean? First, it provides evidence against the origin of the ecotypes during a period of past allopatric separation. An allopatric period was also excluded in the one case of a within-region analysis that favored the old divergence model (Sweden). Thus, the available evidence strongly suggests that the crab and wave ecotypes of *L. saxatilis* were formed by divergent selection in the face of continuous gene flow. The contrasting result for Sweden may reflect more recent common ancestry of the spatially separated sites in that region as a result of postglacial colonization. The two Spanish sites appear to have a long separate history (as observed previously, Quesada et al.2007) and the separation of the British sites may also be older than in Sweden (Panova et al.2011).

Parallel origin, as inferred here, relates to the demographic history of the populations. The inference that ecological barriers between ecotypes developed after geographic barriers between localities does not require that the alleles implicated in the formation of ecological barriers originated independently in each locality. Locally adaptive alleles may have risen in frequency from standing variation present in all founding populations, or may have spread among populations at a later date. Thus, of the options presented by Johannesson et al. (2010) for the origin of parallel local adaptation, the single-origin alternative (scenario A) can be excluded but the different genetic pathways to parallel local adaptation in the presence of gene exchange (B1–B3) cannot easily be separated. The inference of a long lag after colonization before formation of ecotypes argues against an origin from standing variation, which is likely to be rapid (Barrett and Schluter 2008). However, ongoing gene exchange, even among regions, suggests that independent origins of either the same or different alleles are less likely than the sharing of variation ancestrally or via concerted adaptation, which is similar to the process described by Morjan and Rieseberg (2004) and the “transporter hypothesis” of Schluter and Conte (2009). The observed sharing of a few outlier AFLP loci hints at a contribution from concerted adaptation. Data for the arginine kinase locus in a related species, *Littorina fabalis*, also point in this direction (Kemppainen et al.2011). Further study of loci influenced by divergent selection (Wilding et al.2001; Wood et al.2008; Galindo et al.2009, 2010) should provide tests of these predictions, although distinguishing among the alternatives may be impractical if divergence depends on many loci of small effect.

Sambatti et al. (2012) have recently compared direct and indirect estimates of gene flow between sunflower (*Helianthus*) species. Following Strasburg and Rieseberg (2008), they emphasize that low levels of genetic differentiation, implying high *Nm*, may reflect large population sizes rather than high gene exchange (*m*). This is important because divergence under selection requires *s* > *m*, whereas divergence under drift requires low *Nm*. Wood et al. (2008) considered this issue in relation to estimates of *F*_ST_ for loci putatively under divergent selection between crab and wave exposed habitats in Britain. They found that the estimated strength of selection was too high to be compatible with the apparently very small genomic regions of elevated differentiation. Using estimates obtained here (*m* ∼ 10^−6^ between morphs) implies much weaker selection on the outlier loci (*s* ∼ 10^−3^), which is more consistent with the observed genomic pattern of differentiation.

Speciation is typically a protracted process during which many changes in geographic distribution, population size, and opportunity for gene flow are likely to occur (Abbott et al.2013). Gene exchange at later stages may easily obscure the signatures of events occurring earlier in the process, particularly in neutral loci (Via 2009; Bierne et al.2013). Current methods for inferring past patterns of gene exchange have serious limitations (Strasburg and Rieseberg 2013), including the uncertainty inherent in interpreting the results of fitting models that are not accurate reflections of the true history, because of the inevitable need for simplification (Becquet and Przeworski 2009). ABC approaches have greater flexibility than many other methods (Beaumont 2010), allowing us, in this case, to combine information from multiple marker types, to tailor demographic models to our knowledge of the study species and to focus on the specific issue of parallel origin. Nevertheless, they are not free from these very general reservations about historical reconstructions.

We conclude that the *L. saxatilis* ecotypes most likely diverged in the presence of gene flow and are certainly now maintained despite gene flow. The ABC analyses, combining information from multiple markers of different types, suggest that the ecotypes have originated repeatedly in different localities. This provides a firm foundation for understanding the genetic basis of divergent adaptation and the nature of other barriers that impact on patterns of gene flow across the genome.

## DATA ARCHIVING

The doi for our data is 10.5061/dryad.m186r.

## Supporting Information

Figure S1 Haplotype networks derived from mtDNA and nuclear sequence data.

Figure S2 Logistic regressions for model validation.

Figure S3 Posterior quantile analyses used in model checking.

Table S1 Primers and annealing temperatures.

Table S2 Summary statistics for all datasets, as used in the ABC analyses.

Table S3 Parameter estimates from the ABC models.
