# Supplementary figures and images for: PARALLEL EVOLUTION OF LOCAL ADAPTATION AND REPRODUCTIVE ISOLATION IN THE FACE OF GENE FLOW
Source: Evolution. 2013 Dec 23;68(4):935–49. doi: 10.1111/evo.12329 (PMC4261988; doi:10.1111/evo.12329)

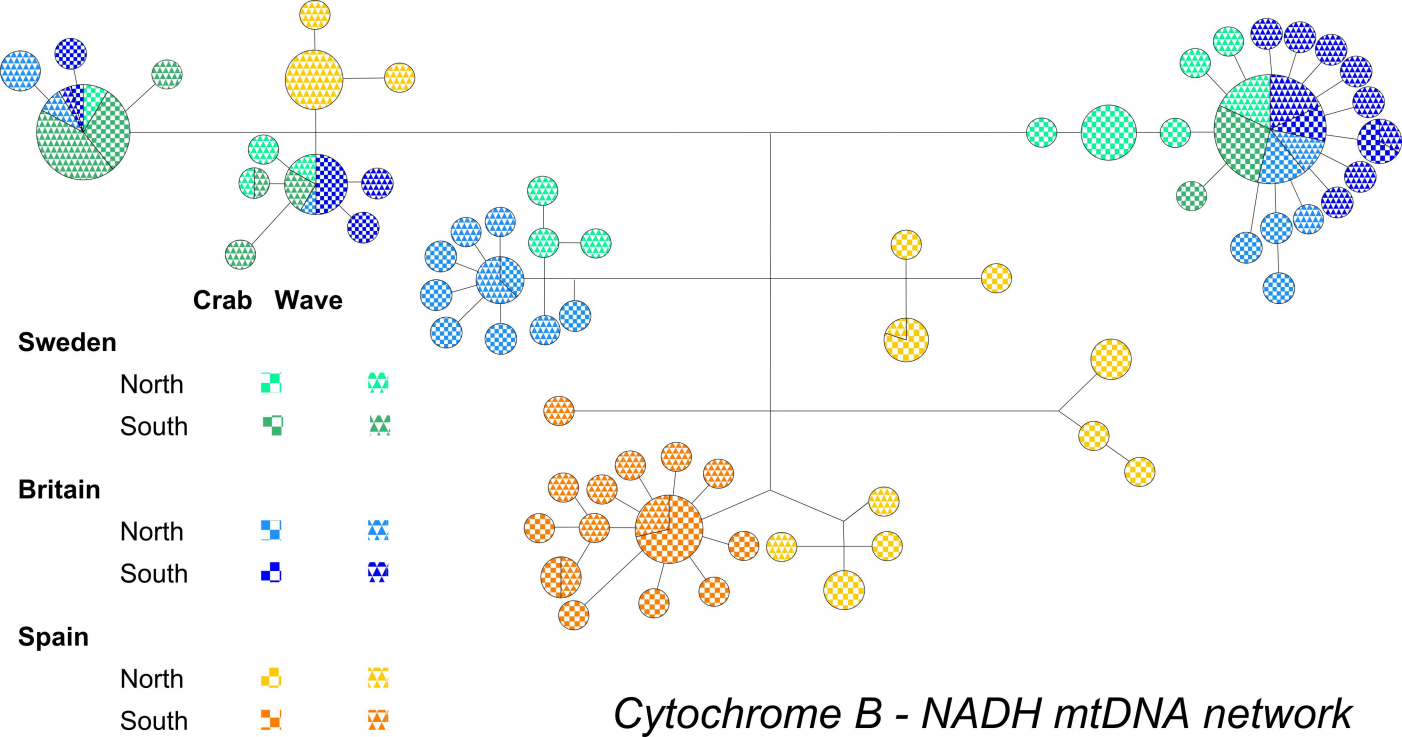

*Cal*

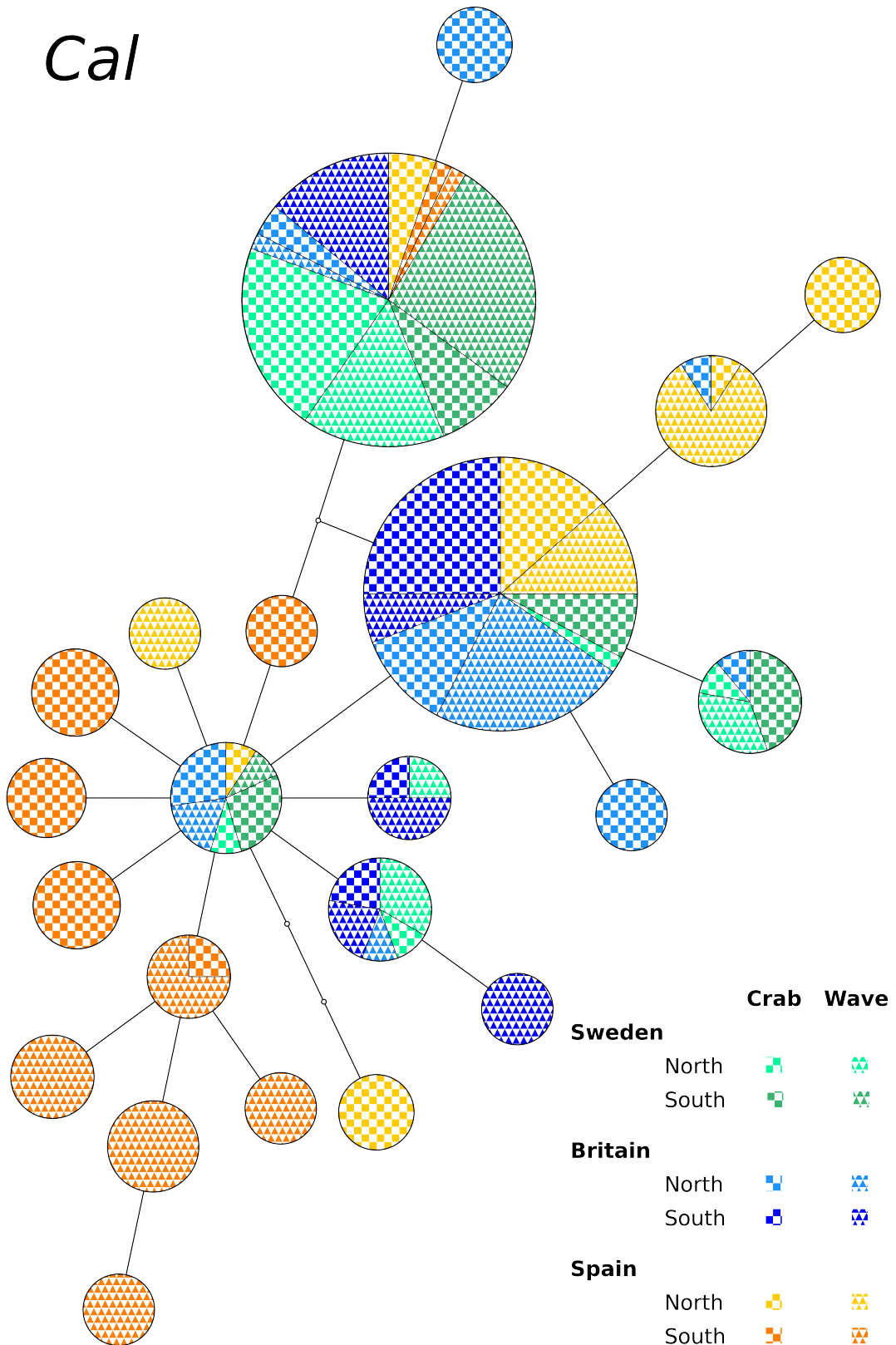

# ElFac

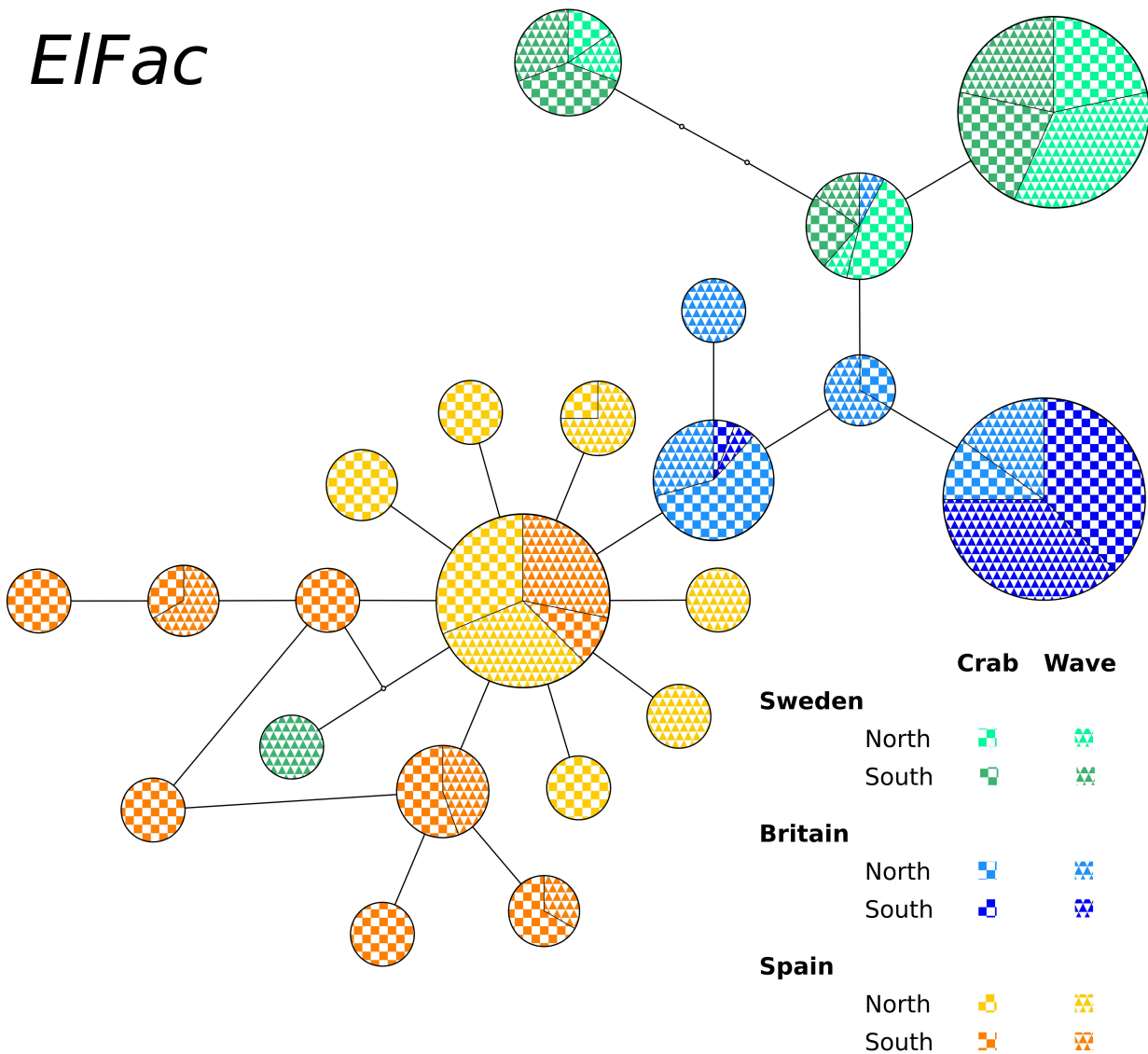

# ThioPer

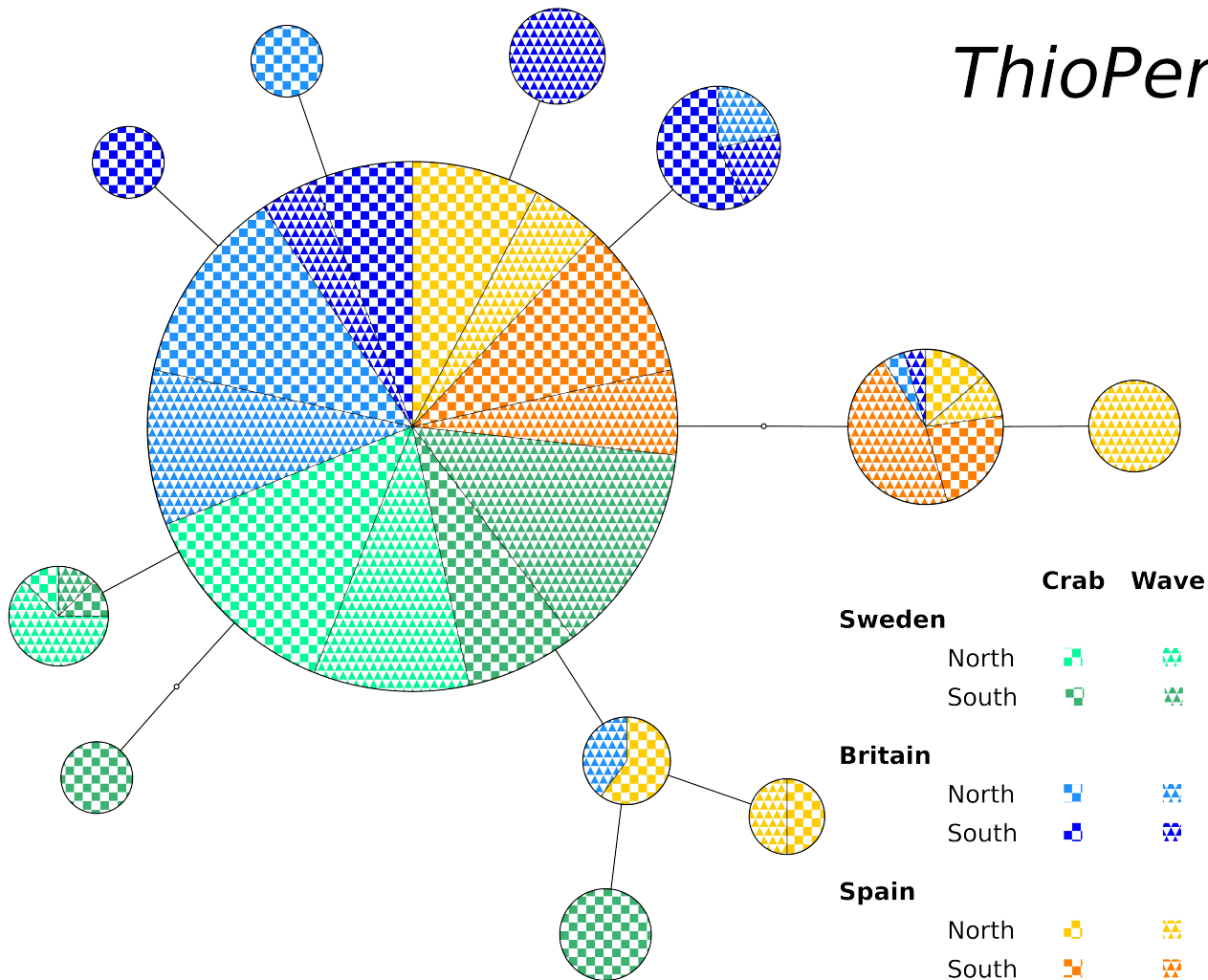

Supplement: Figure S1 — Haplotype networks derived from mtDNA and nuclear sequence data. [file evo0068-0935-SD1.pdf]
