# Supplementary material for: PARALLEL EVOLUTION OF LOCAL ADAPTATION AND REPRODUCTIVE ISOLATION IN THE FACE OF GENE FLOW
Source: Evolution. 2013 Dec 23;68(4):935–49. doi: 10.1111/evo.12329 (PMC4261988; doi:10.1111/evo.12329)

Figure S2. Logistic regressions for model validation: A – within-region, B – between-region. The model under which each pseudo-observed data set was generated is specified by the 'model classifier' (0 – parallel divergence, 1 – old divergence).

We simulated 1000 (new) data sets from the original priors for each competing model. This gave us 2000 parameter sets and the 2000 resulting vectors of summary statistics in total. We took each of the summary statistic vectors in turn as a pseudo-observed data set and tested, using the model choice procedure described above, how often we could identify correctly the model that produced the pseudo-observed data set. We calculated the approximate posterior probability of the "old-divergence model" for each of the pseudo-observed data sets (by dividing the marginal density of the old-divergence model by the sum of the marginal densities of the two models). These values are plotted against the categorical model classifier. A logistic regression (solid line) shows the robustness of discrimination between models.

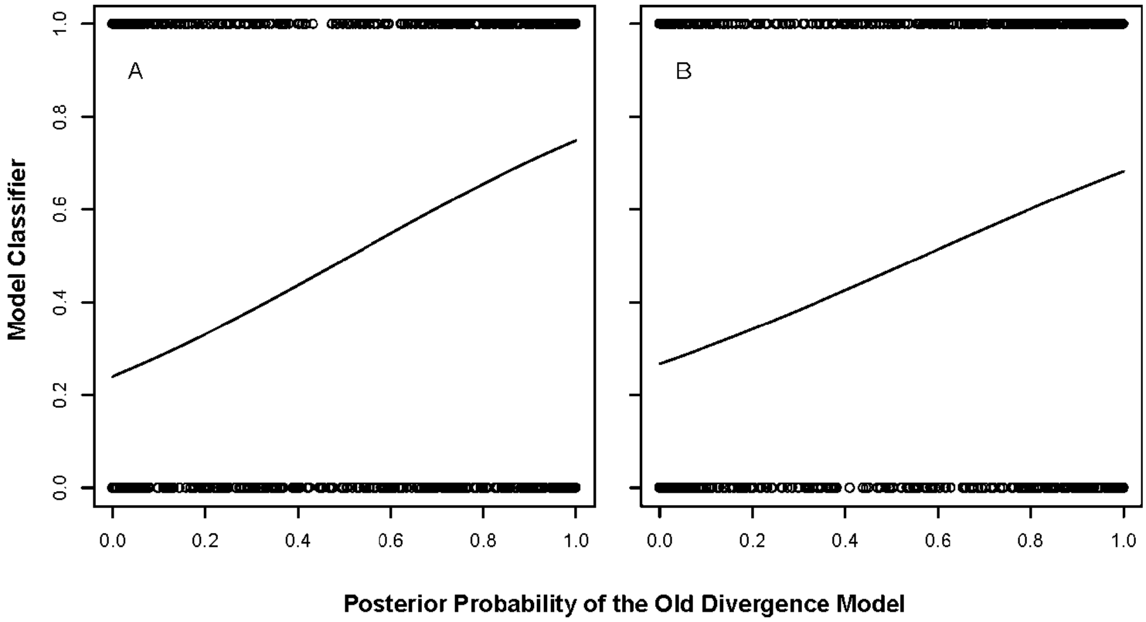

Supplement: Figure S2 — Logistic regressions for model validation. [file evo0068-0935-SD2.pdf]
