# Supplementary material for: PARALLEL EVOLUTION OF LOCAL ADAPTATION AND REPRODUCTIVE ISOLATION IN THE FACE OF GENE FLOW
Source: Evolution. 2013 Dec 23;68(4):935–49. doi: 10.1111/evo.12329 (PMC4261988; doi:10.1111/evo.12329)

Figure S3. Posterior quantile analyses used in model checking.

To validate our parameter estimates, we used the 1000 pseudo-observed data sets that were generated under each model and, using the parameters (drawn from the priors) and the resulting summary statistics as true parameters and pseudo-observed data sets respectively, we looked for potential bias in the posterior distributions (see ABCtoolbox manual, p.48; http://www.cmpg.iee.unibe.ch/content/softwares__services/computer_programs/abctoolbox/index_eng.html). For each parameter of interest, a Kolmogorov-Smirnov test, implemented in R, was used to check for uniformity of the posterior quantiles.

A. Within-region model:

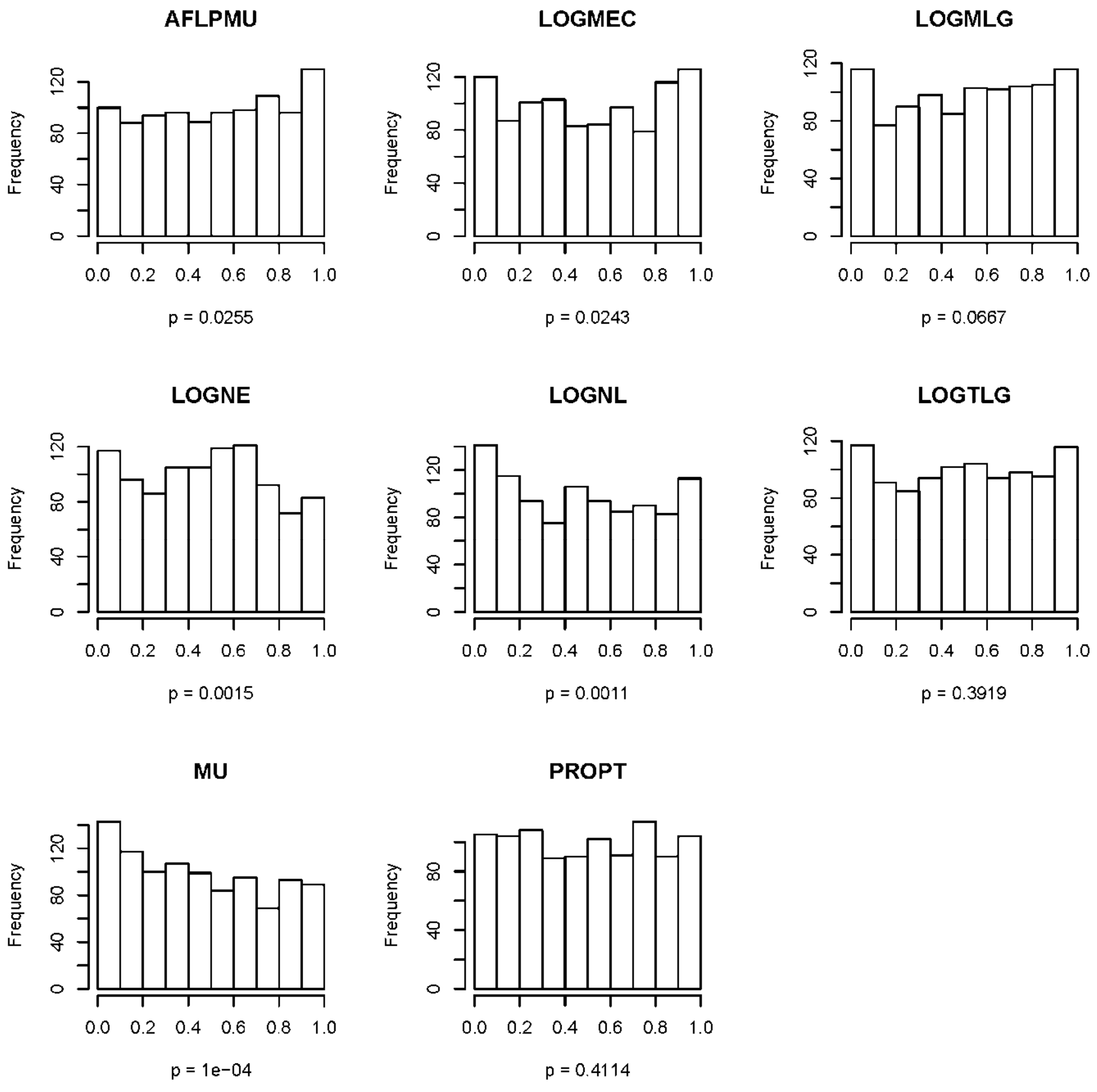

B. **Between-region model**

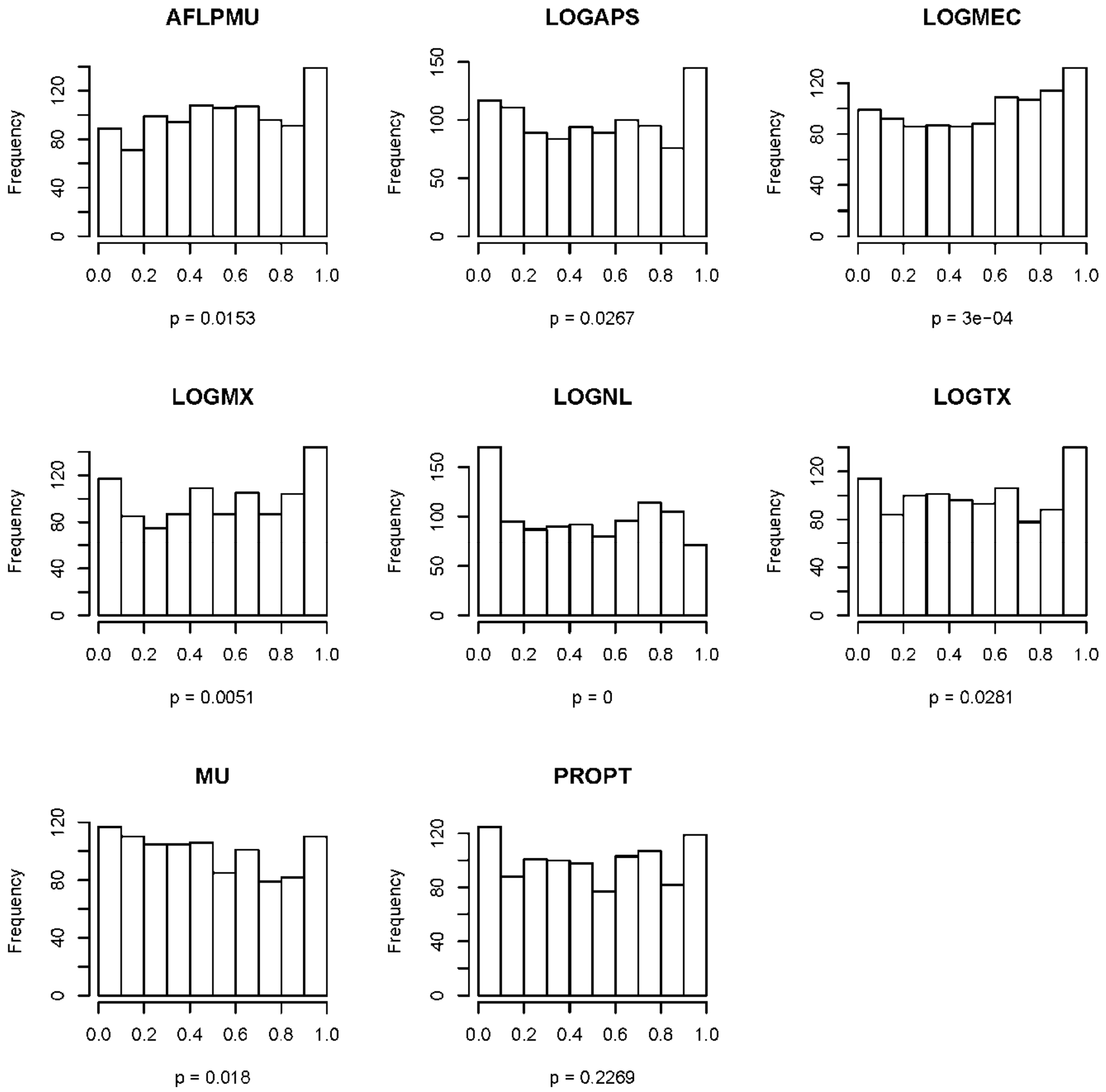

Supplement: Figure S3 — Posterior quantile analyses used in model checking. [file evo0068-0935-SD3.pdf]
