# Supplementary material for: PARALLEL EVOLUTION OF LOCAL ADAPTATION AND REPRODUCTIVE ISOLATION IN THE FACE OF GENE FLOW
Source: Evolution. 2013 Dec 23;68(4):935–49. doi: 10.1111/evo.12329 (PMC4261988; doi:10.1111/evo.12329)
Supplement: Table S1 — Primers and annealing temperatures. [file evo0068-0935-SD4.docx]

**Parallel Evolution of Local Adaptation and Reproductive Isolation in the Face of Gene Flow**

Roger K. Butlin, Maria Saura, Grégory Charrier, Benjamin Jackson, Carl André, Armando Caballero, Jerry A. Coyne, Juan Galindo, John W. Grahame, Johan Hollander, Petri Kemppainen, Mónica Martínez-Fernández, Marina Panova, Humberto Quesada, Kerstin Johannesson and Emilio Rolán-Alvarez

SUPPLEMENTARY MATERIAL

**Table S1** Primers, annealing temperatures (T_a_) and amplification, cloning and sequencing methods

| **Locus** | **Primer name** | **Sequence** | **T_a_ (°C)** |
| --- | --- | --- | --- |
| **MtDNA** | ND-F  ND-R  CYT-F  CYT-R | 5’- GCA GGA CTC CCA CAA CCT TTA G  5’- AGC AGC CCT CCA ACA TAA A  5’- CTG CGC TCT GAA AAT GAC  5´- GGA CTA GGG CCG AAA GTA TAA ATA | **53** |
| ***Cal*** | Cal-F | 5'-AAG CAT GAG CAG AAC ATC GAC | 64 |
|  | Cal-R2 | 5'-TGA CCT CGT AGG TGT TGT CTG |  |
| ***ElFac*** | ElFac-F4 | 5'-TCA AGA AGA TCG GGT ACA ACC C | 65 |
|  | ElFac-R | 5'-ACG TTG AAC CCG ACG TTG TC |  |
| ***ThioPer*** | ThioPer-F2 | 5'-ACA TGA ACA TCC CTC TGA TGG | 65 |
|  | ThioPer-R | 5'-ACA AAC TTC TCC GTG CTT GTC |  |
| **AFLP** |  | Eco+AAC/Pst+ATG Eco+AAC/Pst+AAG Eco+AAC/Pst+AGC Eco+AAC/Pst+ACC Eco+AGG/Pst+ACT Eco+AGG/Pst+AAC Eco+AGA/Pst+ATG Eco+ AGA/Pst+AAG Eco+ AGA/Pst+AGC Eco+ AGA/Pst+ACC Eco+ AGA/Pst+ACT Eco+ AGA/Pst+AAC |  |

For the nuclear loci, T_a_ was 2°C lower than the annealing temperature calculated according to Finnzyme (http://www.finnzymes.fi/optimizing_tm_and_annealing.html)

*Mitochondrial PCR and sequencing*

PCR was carried out with 100ng of extracted genomic DNA, 2.5µL of 10X reaction buffer, 1.5µL 50mM MgCl_2_, 1µL of each primer at 10μM, 1U of Taq DNA polymerase and deionized water up to a final volume of 25 µL. Cycling conditions consisted of 5min denaturation at 95 º C, followed by 30 cycles of 20s denaturation at 95ºC, 20s annealing at 53ºC and 30s extension at 72ºC, followed by a final 7min extension at 72ºC. PCR products were purified using GFX columns (Amersham Biosciences). For each PCR product, both strands were sequenced directly, using the same primers as for the PCR amplification, on an ABI 3730XL sequencer with BigDye chemistry (Applied Biosystems).

*Nuclear intron PCR, cloning and sequencing*

In order to avoid PCR artefacts, all nuclear gene amplifications were performed with the Phusion^®^ High-Fidelity DNA Polymerase (Finnzymes). In addition the amplification protocol was tuned in order to reduce the formation of chimeric sequences ([Lahr and Katz 2009](#_ENREF_1)). The optimized protocol consisted of two successive PCR reactions, performed with an excess of primers and a reduced amount of DNA template. Moreover, for each PCR, the extension step was lengthened to 1 min. 30 s, and the number of cycles was adjusted to 27 and five, respectively, for the first and second PCR. The first PCR was carried out in 13 µL volume, containing 1 X Phusion^®^ HF Buffer, 0.2 mM of each dNTP, 1 µM of each primer, 0.01 U.µL^-1^ of Phusion^®^ DNA Polymerase (Finnzymes), and 1 µL of DNA template diluted at 1/20. The thermocycling regime was set up according to Finnzymes’ recommendations: 98°C for 3 min., (98°C for 10 s, T_a_ for 30 s, 72°C for 1 min. 30 s) x 5 cycles, (98°C for 10 s, T_a_ for 15 s, 72 °C for 1 min. 30 s) x 17 cycles, 72°C for 5 min. The PCR products were checked on 1.5% agarose gels. Then, a “reconditioning” PCR ([Thompson 2002](#_ENREF_4)) was conducted with the same reaction mix as to the first amplification, but with 1 µL of PCR products diluted at 1/5. This second PCR consisted of a very short reaction: 98°C for 3 min., (98°C for 10 s, T_a_ for 30 s, 72°C for 1 min. 30 s) x 5 cycles, 72°C for 5 min.. Finally, the “reconditioned” PCR products were checked on 1.5% agarose gel, and then cloned using the Zero Blunt^®^ TOPO^®^ PCR Cloning Kit (Invitrogen).

For each individual sample, up to 16 colonies were picked per gene, and eluted in 50 µL. The clones were directly amplified in 10 µL volume, containing 1 X Taq Buffer Advanced (with 1.5mM MgCl_2_; Eppendorf 5 Prime), 0.2 mM of each dNTP, 0.275 µM of each primer M13F(-20) (5'-GTAAAACGACGGCCAG) and M13R (5'-CAGGAAACAGCTATGAC), 0.02 U.µL^-1^ of Taq Polymerase (Eppendorf 5 Prime), and 1 µL of template. The thermocycling regime was as follows: 95°C for 10 min., (95°C for 30 s, 50°C for 30 s, 68°C for 30 s) x 35 cycles, 68°C for 7 min.. The clones were then screened on 1.5% agarose gels. At least four positive clones were selected per gene and per sample, and amplified again, using the same PCR protocol, but in 50 µL. The products of this last PCR were checked on 1.5% agarose gels and sent to MACROGEN (www.macrogen.com) for cleaning and sequencing.

*AFLP PCR conditions*

These were performed in 10µl of 1X PCR buffer (ABgene) containing: 2mM MgCl_2_, 0.2mM dNTPs, 0.5µM Eco+A primer, 0.5µM Pst+A primer, 0.25U of Thermoprime Plus *Taq* (ABgene) and 2µl of template. Pre-selective PCRs were diluted 1:8 and used as template for selective PCRs. These were performed in 5µl of 1X PCR buffer (ABgene) containing: 2mM MgCl_2_, 0.2mM dNTPs, 0.5µM Pst+3 primer, 0.075µM Eco+3 (6-FAM) primer, 0.05µM Eco+3 (NED) primer, 0.125U of Thermoprime Plus *Taq* (ABgene) and 1µl of template.

PCR products from selective reactions were electrophoresed along with Genescan ROX 500 (Applied Biosystems) and a 585 bp (ROX) size marker generated in house in an ABI 3730 DNA analyzer (Applied Biosytems). GeneMapper v.3.7 (Applied Biosystems) was used to analyse the AFLP profiles, fragments from 50 to 585 bp were included.

*Dimensionality reduction using PLS*

We reduced dimensionality using PLS components for parameter estimation. We used a script in ABCtoolbox, which uses the PLS package ([Mevik and Wehrens 2007](#_ENREF_2)) in R ([R Core Team 2012](#_ENREF_3)). We used the first 10 components in all instances because further components contributed little to the reduction in root mean squared error of the modelled parameters. Prior to defining the PLS components, we transformed the summary statistics using a Box-Cox transformation, in order to linearize the relationship between the parameters and summary statistics. The 10 components used consistently captured information in the summary statistics (>70% of variance explained in all cases) but the information provided about the model parameters varied ([root mean squared error reduced by 1-40% depending on the parameter and model, see Wegmann et al. 2009](#_ENREF_5)). Population size and time parameters were generally estimated better than migration or mutation parameters.

*Literature cited*

Lahr, D. J. G., and L. A. Katz. 2009. Reducing the impact of PCR-mediated recombination in molecular evolution and environmental studies using a new-generation high-fidelity DNA polymerase. BioTechniques 47:857-866.

Mevik, B.-H., and R. Wehrens. 2007. The pls package: Principal Component and Partial Least Squares regression in R. Journal Of Statistical Software 18:1-24.

R Core Team. 2012. R: A language and environment for statistical computing. R Foundation for Statistical Computing, Vienna, Austria.

Thompson, J. R. 2002. Heteroduplexes in mixed-template amplifications: formation, consequence and elimination by 'reconditioning PCR'. Nucleic Acids Research 30:2083-2088.

Wegmann, D., C. Leuenberger, and L. Excoffier. 2009. Efficient approximate Bayesian computation coupled with Markov chain Monte Carlo without likelihood. Genetics 182:1207-1218.

**Table S2**. Summary statistics for all data sets, as used in the ABC analyses. See separate file.

**Table S3**. Parameter estimates from the ABC models. See separate file.

**Figure S1.** Haplotype networks derived from mtDNA and nuclear sequence data. Pie charts representing haplotype frequencies in each population sample were drawn in LibreOffice (www.libreoffice.org), based on the allele frequencies calculated in Arlequin.
